# Palliative Care Professionals’ Inner Lives: Cross-Cultural Application of the Awareness Model of Self-Care

**DOI:** 10.3390/healthcare9010081

**Published:** 2021-01-15

**Authors:** Amparo Oliver, Laura Galiana, Gustavo de Simone, José M. Tomás, Fernanda Arena, Juan Linzitto, Gladys Grance, Noemí Sansó

**Affiliations:** 1 Department of Methodology for the Behavioral Sciences, University of Valencia, 46010 València, Spain; Amparo.oliver@uv.es (A.O.); Laura.galiana@uv.es (L.G.); Jose.M.Tomas@uv.es (J.M.T.); 2 Pallium Latinoamérica Institute, Buenos Aires 1264, Argentina; ggds55@gmail.com (G.d.S.); juanpamba@gmail.com (J.L.); gmgrance@gmail.com (G.G.); 3 PostDoc Position in Post-Graduate Program in Social Work, School of Humanities, Pontifical Catholic University of Rio Grande do Sul., Porto Alegre 90619-900, Brazil; fernanda.arena@edu.pucrs.br; 4 Department of Nursing and Physiotherapy, University of Balearic Islands, 07122 Palma, Spain; 5 Balearic Islands Health Research Institute (IDISBA), 07120 Palma, Spain

**Keywords:** compassionate care, compassion satisfaction, compassion fatigue, cross-cultural comparison

## Abstract

Compassionate professional qualities traditionally have not received the most attention in either critical or end of life care. Constant exposure to death, time pressure and workload, inadequate coping with personal emotions, grieving, and depression urge the development of an inner curricula of competences to promote professional quality of life and compassionate care. The COVID-19 pandemic highlights the universality of these problems and the need to equip ourselves with rigorously validated measurement and monitoring approaches that allow for unbiased comparisons. The main objective of this study was to offer evidence on the generalizability of the awareness model of self-care across three care systems under particular idiosyncrasy. Regarding the sample, 817 palliative care professionals from Spain, Argentina, and Brazil participated in this cross-sectional study using a multigroup structural equation modeling strategy. The measures showed good reliability in the three countries. When testing the multigroup model against the configural and constrained models, the assumptions were fulfilled, and only two relationships of the model revealed differences among contexts. The hypotheses posited by the awareness model of self-care were supported and a similar predictive power on the professional quality of life dimensions was found. Self-care, awareness, and coping with death were competences that remained outstanding no matter the country, resulting in optimism about the possibility of acting with more integrative approaches and campaigns by international policy-makers with the consensus of world healthcare organizations.

## 1. Introduction

Person-centered care, as a caring philosophy, holds that there is no appropriate healthcare unless it is compassionate [1]. Compassion or “suffering with”[2] has been defined as “a virtuous response that seeks to address the suffering and needs of a person through relational understanding and action”[3]. Moreover, kindness and equanimity are essential qualities in those who care for the dying. However, there is currently a great concern that these compassionate qualities are not always present in the care of the dying [3,4,5]. International studies have highlighted important levels of compassion fatigue in healthcare professionals in general [6,7,8,9], and in palliative care professionals in particular [10,11]. Specifically, in the Spanish context, 69% of nurses and 77% of physicians had, either firsthand or through close colleagues, experienced being the second victim within the following five years [12].

The latest literature focusing on person-centered care delivery considers the preferences, needs, and values of the receivers of these services [13,14,15,16,17,18]. The difficulty in compassionate care is related to several stressors that affect palliative care professionals, such as exposure to death, inadequate time to deal with patients, growing workload and communication difficulties with patients and their families, or inadequate coping with their own emotional response to grieving, depression, and guilt [19,20]. Compassion is also linked to protective factors, such as training in emotional management and spirituality [21,22,23,24,25], self-care [26,27,28,29], empathy [30], awareness [31,32,33,34,35,36], or competency and attitudes toward death [37].

Attending the literature on compassion protectors, specifically based on Kearney and Weininger’s awareness model of self-care [38], Sansó et al. [39] tested a mapping model with variables involved in palliative care professionals’ quality of life: compassion satisfaction (CS), compassion fatigue (CF), and burnout (BO). The other variables included were self-care and awareness, which were positively related to coping with death. Coping together with awareness were posited as being promoting factors of CS due to their positive relationship, and both of them were shown to work as protectors, given their negative relationship with CF and BO [39].

This study, initially carried out in a national sample of Spanish palliative care professionals, is, to the best of our knowledge, the only attempt to study, in a multivariate framework, the inner life of palliative caregivers. This is of paramount importance, as keeping their equanimity, cultivating compassion, and also developing a strengthened sense of vocation and job satisfaction are recognized as key issues in the healing process [39]. However, a major constraint of this study was its rather specific European context, that is, the Spanish one. In order to strengthen the understanding of the stable effects, as well as those that are specific to different cultural contexts, this research was expanded to include other countries, namely, Argentina and Brazil. Finding the dimensions that are protective for the caregiver, the strength they possess, and the specific international circumstances under which they work may guide national policy-makers to make educated improvements to enhance the ability of professional caregivers to provide compassion.

There were two main reasons for the selection of the abovementioned countries. On the one hand, we wanted to test the generalizability of the awareness model of self-care approaching the relationship between specific aspects of professionals’ inner lives through the evaluation of an adaptation of Kearney and colleagues’ awareness model [19,38] in non-European countries. Argentina, while having a very different healthcare system, is one of the few South American countries where hospice and palliative care is widely provided throughout the country [40,41]. On the other hand, it was also of interest to test the model in other countries in which palliative care is still facing several obstacles (e.g., funding, establishment of inclusion criteria, and treatment discontinuity) before its complete integration, such as Brazil [40,42]. This country has a short history of palliative care and many of its services have been recently founded, mainly offering palliative care in hospitals [40,41,43]. Thus, both Argentina and Brazil differ from the Spanish palliative care system. Argentina has achieved “a measure of integration with other mainstream service providers together with wider policy recognition,” as established by Clark and Wright [44].

In this context, the aim of this research was to test the awareness model of self-care, which integrates background and protective variables to explain professionals’ inner lives in terms of quality of life in different countries with different idiosyncratic characteristics in their palliative care attention. For this aim, a multigroup structural equation modeling strategy was used. Compared with other analyses, such as regression analysis, path analysis generates a pictorial representation, which facilitates the interpretation of the model and the hypotheses for the reader, provides means to distinguish effects of one variable from another, and permits standardized errors of the observed variables [45]. In a multigroup context, that is, when studies involve more than one group or population, relationships can vary across these groups, and multigroup models can be used to examine such population heterogeneity [46]. These models study whether the observed variables remain unchanged in different populations—in our case, in the professionals of different countries. The test of equality or invariance of path coefficients across groups enables us to examine similar behavior across groups [47], and therefore, to potentially generalize theories from one group to another.

Our hypotheses are based on Kearney and Weininger’s model [38], whose empirical evidence thus far only exists for Spain [39]:

1. Competence in coping with death and awareness will be positive predictors of CS and negative predictors of CF and BO.
2. Having participated in training programs aimed at facing suffering and death, self-care and awareness will positively predict coping with death, and will indirectly predict professionals’ quality of life (through a mediator effect of coping).
3. The three variables (training, self-care, and awareness) will show positive relationships amongst one another.
4. The dimensions of the professionals’ quality of life will be interrelated: BO will be negatively related to CS and positively related to CF, whereas CS and CF will be independent.

**Figure 1:**
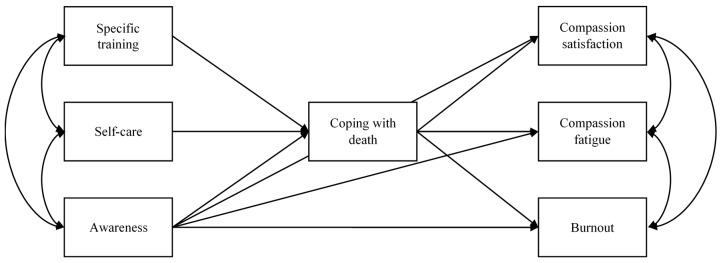
A priori structural model, based on an adaptation of Kearney and Weininger’s model [38], validated by Sansó et al. [39] for the Spanish context.

Figure 1 shows the proposed model.

## 2. Materials and Methods

### 2.1. Design, Procedure, and Participants

The cross-sectional surveys of Spanish, Argentinian, and Brazilian palliative care professionals were conducted between 2013 and 2016. Prior to these surveys, the research protocols were approved by the ethics committees of the professional associations. Members from the Spanish Society for Palliative Care (Spain), the Brazilian National Academy of Palliative Care (Brazil), and the Pallium Latinoamérica Institute, Argentine Association of Medicine and Palliative Care and the National Institute of Cancer (Argentina) were encouraged to participate.

Data were collected using a secure and anonymous online platform, with participation being voluntary and requiring the responders’ informed consent. Regarding the responses, 385 professionals completed the survey in Spain, 271 in Argentina, and 161 in Brazil. The participants’ characteristics are described in Table 1.

There were statistically significant differences among the countries in terms of the mean age (*F*(2.786) = 54.589, *p* < 0.001, *η*2 = 0.12), sex (*χ*^2^(2) = 8.674, *p* = 0.013, Cramer’s *V* = 0.104), and profession (*χ*^2^(10) = 89.331, *p* < 0.001, Cramer’s *V* = 0.233) distribution across samples.

### 2.2. Outcomes

Data were collected using the following measures (internal consistency can be consulted in Table 2):

(a) Specific training in dealing with death and dying [39], measured with a single open-ended question: “Have you done specific training to face suffering and death?”
(b) The Professional Self-Care Scale (PSCS) [48], which assesses three dimensions of professionals’ self-care: physical, which refers to activities that help to maintain a healthy body; inner, which is related to activities that help to keep a healthy mind; social, pertaining to activities related to social activities that help the individual to maintain social health [48].
(c) The Mindful Attention Awareness Scale (MAAS) [49,50], which is a 15-item instrument that measures the general tendency to be aware and conscious of one’s own experiences of daily life.
(d) The Coping with Death Competence Scale, in its Spanish and Portuguese versions [51,52,53], which is composed of 30 items and measures professionals’ mastery when facing death.
(e) The Professional Quality of Life Scale (ProQOL) [54,55], which comprises three subscales: CS, which refers to the positive consequences of helping others; CF, which refers to the negative consequences of helping others; BO, a form of distress manifested by decreased work performance resulting from negative attitudes and behavior.

### 2.3. Data Analysis

The structural models were tested in MPLUS version 8 [56] with maximum likelihood-robust estimation, given the lack of multivariate normality. Firstly, the a priori theoretical model [39] was estimated in the three samples (see Figure 1). Once an adequate fit was obtained for each individual sample, multigroup structural models were stablished in order to test for differences between countries. A multisample strategy was used to test the generalizability of the relationships. The multigroup sequence of models started with a configural or baseline model that had the same relationships but no constraints across groups. Then, a second multisample model was estimated, with all of the structural coefficients in the path model constrained to equality (constrained model). If this constrained model fit the data as well as the baseline model, this would indicate no differences between the samples or, in other words, no moderation effects due to the country. If potential interaction (moderation) effects were found, the modification indices of MPLUS were then used to test the adequacy of releasing each imposed constraint.

Model fit was assessed with chi-square, Comparative Fit Index (CFI), Standardized Root Mean Residual (SRMR), and Root Mean Square Error of Approximation (RMSEA). The following cut-off values were used to determine good fit: CFI above 0.90 and SRMR or RMSEA below 0.08. As a multisample context was used, the models were also comparatively assessed using the chi-square difference test (with no statistical differences meaning preference for the most constrained model) and CFI differences (with differences of 0.05 or less considered negligible) [57].

Missing data were dealt with using the full information maximum likelihood (FIML), which is adequate for both missing completely at random (MCAR) and missing at random (MAR) data and is the most recommended method for structural models [58].

We used the STROBE cross-sectional checklist when writing our report [59]. The research protocol received ethical approval from the Pallium Latinoamérica Institute (code 210316).

## 3. Results

Descriptive statistics for the variables included in the awareness model of self-care can be consulted in Table 2. In general, the means were medium–high for self-care, with higher means for the Spanish group. Moreover, high levels of awareness were found, with higher means for the Argentinian professionals. High levels of coping with death were also found, with higher scores for the Spanish professionals, as were high levels of compassion satisfaction, with higher levels for the Brazilian group. Lastly low–medium levels of compassion fatigue and burnout were found, with higher levels of compassion fatigue for the Spanish and Argentinian samples, and higher levels of burnout for the Brazilian professionals.

The model was independently tested in the samples and fit indices were adequate (see Table 3). Regarding the RMSEA, its performance has proved to be poor in small samples (as in the Brazilian case) and in models with small degrees of freedom, such as the tested model (six degrees of freedom) [60]; however, our appreciation of the overall goodness of fit of the three samples should not change, despite this particular value.

Once the adequacy of the model in each sample was established, the baseline model was tested. This model had no constraints across groups; all of the parameters were freely estimated and simultaneously tested in the three samples. This model showed a good fit (Table 3). Then, a model with all of the parameters constrained across the three samples was estimated, i.e., the fully constrained model. This model was the most parsimonious one, as only the Spanish sample was used for the estimation, whereas the estimates for the Argentinian and Brazilian samples were fixed to these first estimates. As Table 3 shows, the model fit was degraded: using a statistical criterion, the chi-square differences were statistically significant; using a subjective criterion, the CFI differences were within the limit put forward by Little [57]. These results provide evidence of some moderation effects of the country. In order to study these effects, modification indices were considered and the relationships penalizing the model’s fit were released.

The modification indices pointed to two constrains that, when released, improved the model’s fit: the effect of specific training on coping with death in the Spanish sample and the relationship between CS and BO in the Brazilian sample. After releasing these constraints, the chi-square of this last model showed no statistically significant differences to the baseline model, as well as an irrelevant difference of 0.003 between CFIs (see Table 3). Consequently, the model was retained as the most parsimonious one.

**Figure 2:**
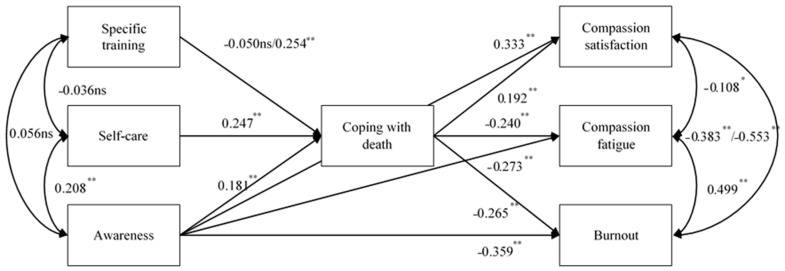
The most parsimonious model with the standardized parameter estimates. Notes: * *p* < 0.050 and ** *p* < 0.001. The non-invariant parameter estimates are those that are duplicated. The first value in the relationship between specific training and coping with death refers to the Spanish sample; the second is that of the Argentinian and Brazilian samples. The first value in the relationship between compassion satisfaction and burnout is for the Spanish and Argentinian samples; the second one is for the Brazilian sample. For the sake of clarity, standard errors are not shown.

The parameter estimates offered evidence of a moderation effect on the relationship posed between specific training and coping with death for the Spanish palliative care professionals. As shown in Figure 1, when compared to the other countries, Spanish palliative care professionals’ specific training had no effect on coping with death, whereas this training had a positive effect for both Argentinian and Brazilian professionals. As regards the second released parameter regarding the relationship between CS and BO in the Brazilian sample, the estimates pointed to a greater relationship in this sample when compared to the Spanish and Argentinian professionals. Both estimates were, however, negative and statistically significant, as hypothesized. All parameter estimates, either invariant or variant, are shown in Figure 2.

Another important result was the considerable and homogeneous amount of variance explained by the most parsimonious model across the three sociocultural contexts (see Table 4). The variance for coping with death ranged from 11.5% (Spain) to 20.8% (Argentina). The protective variables allowed for almost 25% of the prediction of BO, no matter the country. When focusing on countries, a higher explicative power was reached for Argentina.

## 4. Discussion

Person-centered palliative care is largely based on the attention of compassionate professionals. Despite its practical relevance, the recent literature claims compassionate qualities are not always present in professionals when working with patients at the end of their life and their families [3,4,5]. In the last decade, few theoretical approaches have tried to explain the reasons for this lack of professional competence [19,20,34], and empirical evidence based on these models, although robust, is yet limited to a particular European healthcare system [39].

The aim of the present research was to investigate the generalizability of the model tested by Sansó et al. [39] in Spanish professionals of palliative care in two additional countries, namely, Argentina and Brazil. By testing a multigroup model, we evidenced the different effects of one variable on another and how these effects vary across our studied groups [46], and we pointed to generalizations in behavior patterns across populations [47]. For such generalization purposes, we used evidence gathered in the previous literature. The model was mostly based on Sansó et al.’s work, although it included some improvements regarding professionals’ inner life appraisal: self-care was assessed with all of the items of the Professionals’ Self-Care Scale, and awareness was assessed with a shorter and more discriminant measure [50].

The results supported Hypothesis 1: “Competence in coping with death and awareness will be positive predictors of compassion satisfaction and negative predictors of compassion fatigue and burnout.” Both competence in coping with death and awareness promoted higher levels of compassion satisfaction and worked as protectors of compassion fatigue and burnout, with negative relationships with these two last constructs. These two relationships, competence in coping with death and quality of professional life and awareness with professional quality of life, have been well documented in the literature [34,37], although this is the first time they have been tested in several countries.

Regarding Hypothesis 2, “Having participated in training programs aimed at facing suffering and death, self-care and awareness will positively predict coping with death, and indirectly will predict professionals’ quality of life (through a mediator effect of coping),” the results provided evidence on all of the assumed relationships, particularly between specific training and coping with death. The findings revealed that, while in Brazil and Argentina this relationship is significant, it is not in Spain. This lack of an effect of specific training on coping with death was already found in the Spanish sample studied by Sansó et al. [39]. Although the indicator used was the same in the three countries, “Have you done specific training to face suffering and death?,” a possible explanation of the absence of an effect in the Spanish context could be the amount of courses healthcare professionals attend in this country. It is common for palliative care professionals to engage in a vast amount of training throughout their professional lives. This, together with the fact that we investigated an “older” sample, especially in terms of professional experience, could have made the question less discriminant in Spain. An additional result was the one offered by Hypothesis 2a, “These three variables will show positive relationships among one another,” which was supported across the countries.

Finally, Hypothesis 3, “The dimensions of the professionals’ quality of life, that is, compassion satisfaction, compassion fatigue, and burnout, will be interrelated. Burnout will be negatively related to compassion satisfaction and positively related to compassion fatigue, whereas compassion satisfaction and fatigue will be independent,” was also sustained by the model, which also offered additional interesting context-dependent information. There was a stronger relationship between compassion satisfaction and burnout in Brazil compared to the other countries.

To summarize, our results highlighted the model’s generalizability, showing that the key elements of professionals’ inner lives, such as self-care, awareness, or coping with death, are competences that remain outstanding no matter the country, which suggests the convenience of being universally encouraged. On the contrary, two relationships could not be generalized: the lack of a predictive effect of specific training in the Spanish context of palliative care, and the negative relationship between compassion satisfaction and burnout, which was stronger in Brazil than in Spain and Argentina.

The Global Atlas for Palliative Care [61] indicates higher rates for adults in need of palliative care at the end of their life in the European and Western Pacific regions. Latin American countries show lower rates. Indeed, European and Western Pacific professionals of palliative care work with elderly patients, in comparison to Latin American professionals, where the end of life is a more natural path for younger professionals. This, however, did not affect the majority of the relationships specified in the current research.

The maturity of the palliative care system is another characteristic that could explain differences in the functioning of the model. The biggest variance accounted for by coping with death, satisfaction and fatigue compassion, and burnout, being explained by protectors in Argentina, could be partially understood by their major efforts in developing palliative professionals’ inner curricula during the last decade. In addition, the Argentinean palliative system has encouraged specific training due to the role played by the *Pallium Latinoamérica* Institute [62]. If we focus on Latin America, clear differences arise in the palliative care contexts, as Argentinian palliative institutions emerged in the early 1980s, whereas in Brazil, they did not emerge until the late 1990s, with the main association (*Academia Nacional de Cuidados Palitivos,* ANCP) being created in 2005 [41]. Chile, Costa Rica, Argentina, and Uruguay pioneered palliative care in this area; Brazil, and other countries such as Colombia, Mexico, and Paraguay, are in a medium state of development, while countries such as Honduras, Nicaragua, and Bolivia are the most delayed in this development. The Brazilian palliative care context is especially interesting for three main reasons: (a) professionals work with younger patients than in Europe; (b) they work in a context of great care discontinuity, as home care initiatives are not integrated in primary healthcare services [43], as it is the case in Spain; (c) caregivers’ quality of life is strongly affected by the difficulties in home care and work overload because not only do professionals provide medical assistance in hospitals, but they also have to work together with the home-care team [63,64]. A more mature palliative care system would bring higher funding, more specific inclusion criteria, treatment continuity, better integration with other mainstream services, and wider policy recognition for those countries with a great tradition in this care. Moreover, the models did not significantly differ, and thus factors protecting professionals from burnout and compassion fatigue and promoting compassion satisfaction seem non-dependent on how well-established the provision of palliative care is.

Regarding the practical implications of this study, the findings evidence the fact that the practice of self-care, the development of awareness, and specific training enhance professionals’ inner lives, directly influencing their quality of life and likely the quality of their caregiving. Working on the variables that increase professionals’ quality of life, a double objective can be achieved: professional wellbeing can be improved (understood as the presence of high compassion satisfaction and low burnout and compassion fatigue), and professionals’ efficacy as healing agents in the palliative care encounter can be optimized through an enhanced ability to use themselves as healing agents in clinical encounters [32].

This study presents some limitations to bear in mind. The first limitation is the low response rate of this kind of study, with a non-incentivized self-report questionnaire. Despite such difficulties, the sample size obtained provided a robust dataset to explore the validity of the awareness model of self-care in different countries with different idiosyncratic characteristics in their palliative care attention. Secondly, it is worth noting that the possibility of response bias is present. To reduce the likelihood of such a bias, the respondents were informed that the research was anonymous.

## 5. Conclusions

This study highlights both the similarities and differences across palliative care professionals of different populations. Such similarities in behavior patterns have been assumed many times but were tested in this study for the first time. Therefore, this study offers evidence of the ability to generalize scientific evidence, including the importance of self-care, awareness, and coping with death for palliative care professionals in different parts of the world.

In conclusion, the contribution of this work is its provision of the first cross-cultural evidence (including two languages and three countries) on the suitability of a comprehensive model to address the relationship between protectors and quality of work life, as well as its quantification of the relationships in the model so that policy-makers can prioritize actions. The benefits from recent interventions in contexts, such as palliative care, with high emotional demands to promote professional quality of life are very encouraging [65,66] and are well structured [67].

In light of our results, even when healthcare systems are not mimetic and show great differences, the protectors of professionals’ quality of life are the same and have the same quantitative effect. That is, the model is generalizable across countries and health systems. This is of special importance, taking into account that preventing burnout and compassion fatigue and enhancing compassion satisfaction are a requisite for both the quality of patients’ care and occupational safety. Compassion is key to meeting patients’ needs, including those on the surface as well as those kept more hidden, and is also crucial for institutional benefits. Compassionate professionals are able to work more and work to a better standard, and, most importantly, can provide more and better-quality care. Thus, interventions attending to the predictors of professionals’ quality of life, such as mindfulness-based stress reduction interventions or compassion-based training, must be on the agenda of world health agencies and policy-makers from now on.

## Figures and Tables

**Table 1:** Sociodemographic characteristics of the participants.

Variables and Categories	Spain		Argentina		Brazil	
	M	SD	M	SD	M	SD
Age	46.8	8.87	43.41	9.69	37.22	11.1
Palliative care experience (years)	10.69	6.59	7.95	5.85	4.97	4.42
	*N*	%	*N*	%	*N*	%
Sex						
Women	297	77.1	214	79.9	141	87.6
Men	85	22.1	51	18.8	18	11.2
Missing	3	0.8	6	2.2	2	1.2
Profession						
Doctors	168	43.6	136	50.2	35	21.7
Nurses	128	33.2	39	14.4	31	19.3
Psychologists	55	14.2	43	15.9	40	24.8
Assistant nurses	15	4.1	3	1.1	0	0.0
Social workers	19	4.9	21	7.7	19	11.8
Other professions	0	0.0	22	8.1	33	20.5
Missing	0	0.0	7	2.6	3	1.9

Notes: M = mean; SD = standard deviation.

**Table 2:** Estimates of the internal consistency, descriptive statistics, and missing data for the variables under study.

Variables and Indicators	Cronbach’s Alphas			Means (SD)			Respondents (Missing Data)		
Variables	S	A	B	S	A	B	S	A	B
Self-Care	0.78	0.76	0.79	3.44 (0.84)	3.26 (0.92)	3.33 (0.91)	356 (29)	258 (13)	145 (16)
Awareness	0.90	0.88	0.83	4.71 (0.88)	4.75 (1.02)	4.60 (0.99)	352 (33)	258 (13)	125 (36)
Coping with death Competence	0.89	0.89	0.92	5.33 (0.80)	5.22 (0.85)	5.04 (1.05)	383 (2)	252 (19)	130 (31)
Compassion satisfaction	0.77	0.86	0.78	5.01 (0.51)	5.05 (0.55)	5.17 (0.65)	329 (44)	239 (32)	121 (40)
Compassion fatigue	0.78	0.77	0.75	2.57 (0.52)	2.57 (0.64)	2.50 (0.64)	385 (0)	240 (31)	121 (40)
Burnout	0.54	0.65	0.68	2.26 (0.58)	2.29 (0.63)	2.42 (0.66)	329 (56)	240 (31)	121 (40)
	**Frequency, Yes (%)**			**Frequency, No (%)**			**Frequency, Missing Data (%)**		
**Indicators**	**S**	**A**	**B**	**S**	**S**	**S**	**S**	**A**	**B**
Specific training	320 (83.1%)	185 (67.8%)	67 (41.6%)	60 (15.6%)	7 (1.8%)	7 (1.8%)	5 (1.3%)	12 (4.4%)	31 (19.3%)

Notes: S = Spain; A = Argentina; B = Brazil.

**Table 3:** Fit indices of the multisample path analyses.

	*χ* ^2^	df	*p*	CFI	RMSEA	RMSEA CI	SRMR	Δ*χ*^2^	Δdf	*p*	ΔCFI
Model in Spain	27.888	6	<0.001	0.950	0.097	0.063–0.135	0.039	-	-	-	-
Model in Argentina	40.636	6	<0.001	0.914	0.147	0.106–0.192	0.053	-	-	-	-
Model in Brazil	10.335	6	0.111	0.969	0.074	0.000–0.149	0.051	-	-	-	-
Configural model	75.312	18	<0.001	0.928	0.110	0.085–0.137	0.046	-	-	-	-
Constrained model	145.229	48	<0.001	0.878	0.088	0.072–0.105	0.107	72.438	30	<0.001	0.050
Most parsimonious model	104.772	45	<0.001	0.925	0.071	0.054–0.089	0.085	33.767	27	0.173	0.003

Notes: CFI = Comparative Fit Index; RMSEA = Root Mean Square Error of Approximation; RMSEA CI = RMSEA 90% confidence interval; SRMR = Standardized Root Mean Residual.

**Table 4:** Variance accounted for by the coping with death and professional quality of life dimensions across countries based on the R^2^ values.

Variables	Spain	Argentina	Brazil
Coping with death	0.115	0.208	0.133
Compassion satisfaction	0.241	0.259	0.208
Burnout	0.243	0.244	0.244
Compassion fatigue	0.162	0.194	0.186

**Notes:** Results from the best fit, most parsimonious model.

## Data Availability

The data that support the findings of this study are available from the corresponding author, Noemí Sansó, upon reasonable request.
